# BCL-W is a regulator of microtubule inhibitor-induced mitotic cell death

**DOI:** 10.18632/oncotarget.9586

**Published:** 2016-05-25

**Authors:** Shan Huang, Rui Tang, Y.C. Poon Randy

**Affiliations:** ^1^ Division of Life Science, Center for Cancer Research, and State Key Laboratory of Molecular Neuroscience, Hong Kong University of Science and Technology, Clear Water Bay, Hong Kong

**Keywords:** apoptosis, BCL-2, microtubule, mitosis

## Abstract

Microtubule inhibitors including taxanes and vinca alkaloids are among the most widely used anticancer agents. Disrupting the microtubules activates the spindle-assembly checkpoint and traps cells in mitosis. Whether cells subsequently undergo mitotic cell death is an important factor for the effectiveness of the anticancer agents. Given that apoptosis accounts for the majority of mitotic cell death induced by microtubule inhibitors, we performed a systematic study to determine which members of the anti-apoptotic BCL-2 family are involved in determining the duration of mitotic block before cell death or slippage. Depletion of several anti-apoptotic BCL-2-like proteins significantly shortened the time before apoptosis. Among these proteins, BCL-W has not been previously characterized to play a role in mitotic cell death. Although the expression of BCL-W remained constant during mitotic block, it varied significantly between different cell lines. Knockdown of BCL-W with siRNA or disruption of the *BCL-W* gene with CRISPR-Cas9 speeded up mitotic cell death. Conversely, overexpression of BCL-W delayed mitotic cell death, extending the mitotic block to allow mitotic slippage. Taken together, these results showed that BCL-W contributes to the threshold of anti-apoptotic activity during mitosis.

## INTRODUCTION

Mitotic entry is driven by CDK1 and its activator cyclin B1. Conversely, destruction of cyclin B1 by APC/C-dependent ubiquitination provides a cue for mitotic exit. As APC/C activation occurs only after the spindle-assembly checkpoint is satisfied, agents that attenuate microtubules depolymerization (e.g. taxanes) or polymerization (e.g. vinca alkaloid) promote cell cycle arrest in mitosis [1]. Other emerging anticancer chemicals targeting mitotic regulators such as Aurora A, PLK1, or Eg5 (KIF11) also act by triggering protracted mitotic arrest [2].

The fate of cells after protracted mitotic block varies greatly between different cell lines as well as between individual cells from the same cell line [3]. The current model of how the cell fate is determined is based on two stochastically competing networks, one controlling mitotic slippage and the other mitotic cell death. Whether a cell dies or undergoes slippage during a prolonged mitotic block is the consequence of whether the cell first breaches the threshold of cell death or mitotic slippage, respectively.

During mitotic slippage, cells exit mitosis without proper chromosome segregation and cytokinesis. The underlying mechanism appears to be a slow but continuous degradation of cyclin B1 during the mitotic block [4]. The molecular basis of mitotic cell death remains incompletely understood. Although it appears to be operated mainly through intrinsic pathway of apoptosis [5], there is also evidence of mitotic cell death through necrosis [6].

Mitotic cell death is likely to be caused by a combination of an accumulation of apoptotic activators or a loss of apoptotic inhibitors. There is evidence that cyclin B1–CDK1 plays a central role in generating mitosis-specific death signals. Several players of the apoptotic pathway, including survivin [7], caspases, and BCL-2 family of proteins [8] are known to be regulated by cyclin B1–CDK1. Members of the BCL-2 family are particularly interesting in this regard because they contribute to both pro- and anti-apoptotic signaling.

Functionally, members of BCL-2 family can be divided into initiators, effectors, and anti-apoptotic proteins [9]. The initiators (including BAD, BID, BIK, BIM, BMF, HRK, PUMA and NOXA) are BH3-only proteins that transduce pro-apoptotic signals by either neutralizing anti-apoptotic BCL-2 proteins or by directly activating pro-apoptotic effectors. Both effectors (BAX, BAK and probably BOK) and anti-apoptotic proteins are multi-BH domain proteins with similar globular structures. The pro-apoptotic function of the effectors is mediated through the induction of mitochondrial outer membrane permeabilization (MOMP), which is antagonized by anti-apoptotic BCL-2 proteins. At least six members of anti-apoptotic BCL-2 proteins are present in human: BCL-2, BCL-XL (BCL2L1), BCL-W (BCL2L2), MCL-1 (BCL2L3), A1 (BCL2L5), and BCL-B (BCL2L10).

While the underlying principles of CDK1-mediated toxicity remain to be fully understood, several members of the anti-apoptotic BCL-2 proteins have been identified to be targets of cyclin B1–CDK1. BCL-2 is phosphorylated at Ser70 by cyclin B1–CDK1 [10–13]. This alters the flexible loop in BCL-2 to enhance the binding to BAK and BIM and therefore contributes anti-apoptotic activities during mitotic block [14]. BCL-XL is also phosphorylated by cyclin B1–CDK1, leading to the inactivation of its anti-apoptotic activity [15, 16]. Finally, phosphorylation of MCL-1 by cyclin B1–CDK1 induces rapid degradation of MCL-1 in an APC/C^CDC20^-dependent manner [17, 18]. The contribution of other anti-apoptotic BCL-2 proteins to mitotic cell death has not been reported.

Here we studied the role of all the members of the anti-apoptotic BCL-2 family in mitotic cell death. A major challenge for studying mitotic cell death using endpoint analyses is that they do not provide information on the history of the cell before cell death. Live-cell imaging was used in this study to obtain temporal information on the contribution of different anti-apoptotic BCL-2-related proteins on mitotic cell death.

## RESULTS

### Monitoring microtubule inhibitor-induced cell fates

**Figure 1:**
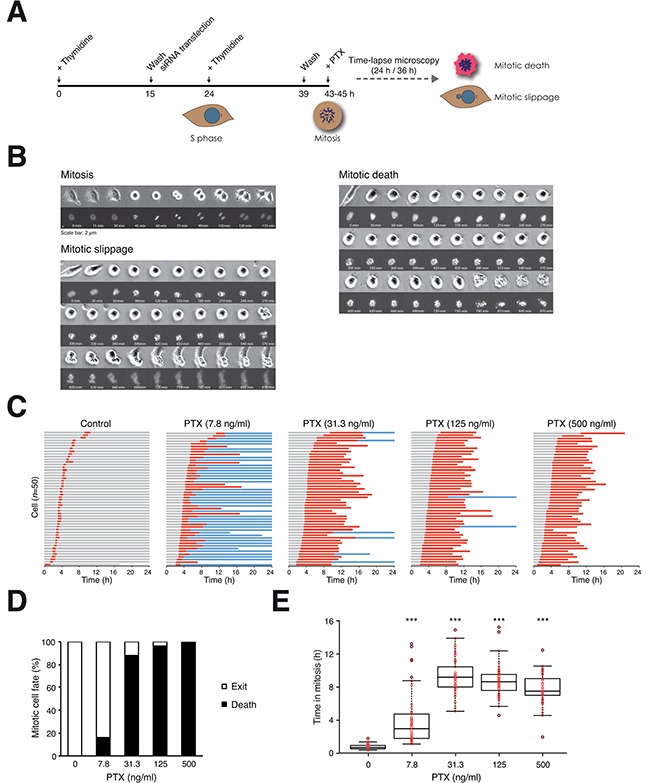
Paclitaxel induces mitotic cell death in a concentration-dependent manner **A.** Schematic diagram of the experimental procedure. The effects of knockdown of BCL-2-like proteins on PTX-induced mitotic cell death were evaluated using live-cell imaging. HeLa cells expressing histone H2B-GFP were synchronized at G_1_/S with a double thymidine procedure and released into the cell cycle for 4 h (HeLa) or 6 h (HCT116) before adding PTX. Individual cells were tracked using live-cell imaging for 24 h (HeLa) or 36 h (HCT116). Only cells entering mitosis during the imaging period were analyzed. All live-cell imaging experiments in this study were performed with this strategy unless stated otherwise. **B.** Examples of normal mitosis, mitotic cell death, and abnormal mitotic exit (slippage) from live-cell imaging of PTX-treated cells. Both bright field (upper panels) and histone H2B-GFP (lower panels) are shown. The complete videos can be found in the Supplemental Materials. **C.** PTX-mediated mitotic cell death. Live traces of HeLa cells (expressing histone H2B-GFP) exposed to either DMSO or a 4-fold increasing concentration of PTX for 24 h (*n*=50). Key: interphase = grey; mitosis (from DNA condensation to anaphase) = red; interphase after mitotic slippage = blue; truncated bars = cell death. **D.** Percentage of different mitotic fates from (C). **E.** Box-and-whisker plots showing elapsed time between mitotic entry and mitotic cell death/exit from (C). One-way ANOVA ***P<0.001 (all compare to DMSO control).

Microtubule inhibitor-induced mitotic block results in multiple cell fates, including mitotic cell death and slippage. To obtain unbiased and temporal information on the different cell fates, we synchronized cells expressing histone H2B-GFP at G_1_/S and released them into the cell cycle in the presence of paclitaxel (PTX), before individual cells were tracked using live-cell imaging (a schematic diagram of the approach is shown in Figure 1A). Examples of time-lapse images of cells undergoing normal mitosis, PTX-mediated mitotic cell death and mitotic slippage are shown in Figure 1B.

Mitotic cell fates were affected by the concentrations of PTX (Figure 1C). As expected, DMSO-treated control cells underwent mitosis synchronously. Incubation with PTX did not affect the timing of mitotic entry. In the presence of a relatively low concentration of PTX, the majority of cells exited mitosis (mainly by mitotic slippage). In contrast, the cell fate switched to cell death at higher concentrations of PTX (Figure 1D). As revealed by the live traces of individual cells (Figure 1C), PTX-induced an increase in both the duration of the mitotic block and the percentage of cells undergoing mitotic cell death (quantified in Figure 1E).

Similar analysis using HCT116 cells revealed that they responded to PTX differently from HeLa. At the same range of PTX concentrations, HCT116 cells mainly underwent mitotic slippage instead of cell death (Supplementary Figure S1). In this study, HeLa and HCT116 were used as representative models mainly undergoing mitotic death and mitotic slippage, respectively.

### Apoptosis accounts for the majority of mitotic cell death

**Figure 2:**
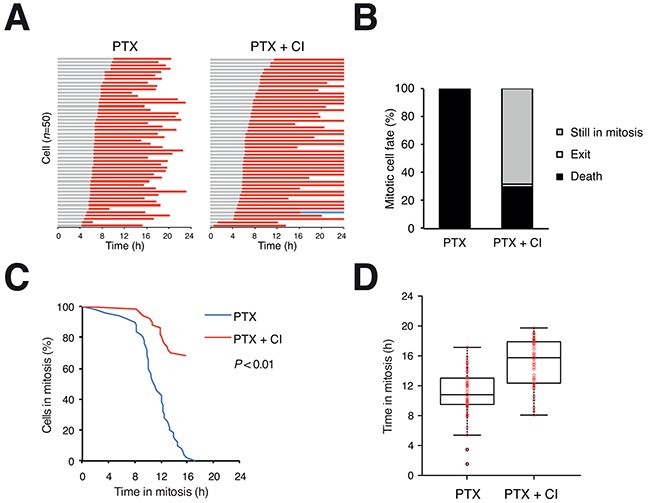
Apoptosis accounts for the majority of microtubule inhibitor-mediated mitotic cell death **A.** Mitotic cell death can be delayed with a pan-caspase inhibitor. Live trace of HeLa cells exposed to PTX (500 ng/ml) in the presence or absence of Z-VAD-FMK (CI) for 24 h (*n*=50). Key: same as Figure 1C. **B.** Percentage of different mitotic fates from (A). In the presence of the caspase inhibitor, more than 60% of cells were still in mitosis at the end of the imaging period. **C.** The durations of mitotic block of cells from (A) are plotted using Kaplan-Meier estimator. **D.** Box-and-whisker plots showing elapsed time between mitotic entry and mitotic cell death from (A).

A pan-caspase inhibitor significantly delayed PTX-mediated mitotic cell death in HeLa cells (Figure 2A). In contrast to the 100% mitotic cell death in PTX-treated cells, more than 60% of PTX- and caspase inhibitor-treated cells were still blocked in mitosis at the end of the 24 h imaging period (Figure 2B) and the mitotic duration was significantly increased (Figure 2C, 2D). Similarly results were obtained with HCT116 cells: although the majority of PTX-treated HCT116 cells underwent mitotic exit (slippage), inhibition of apoptosis abolished the remaining 30% of mitotic cell death (Supplementary Figure S1B). Inhibition of apoptosis was confirmed by detecting PARP1 cleavage (Supplementary Figure S1D).

In contrast to inhibitors of caspases, inhibitors of necroptosis (necrostatin-1) or autophagy (chloroquine) exerted little effect on the timing of mitotic cell death (Supplementary Figure S2). Taken together, these results indicate that apoptosis accounts for the majority of mitotic cell death induced by PTX.

### Selective members of anti-apoptotic BCL-2 family contribute to the kinetics of mitotic cell death

Given that PTX-induced mitotic cell death involves apoptosis, we next investigated which anti-apoptotic BCL-2 family members are involved suppressing mitotic cell death. The mRNAs of individual members of the family could be downregulated with siRNAs by at least 60% (Supplementary Figure S3A). The exception is BCL-B, of which the mRNA was undetectable in both HeLa and HCT116 cells; and hence was not included in further studies. Downregulation at the protein level was also confirmed using immunoblotting (Supplementary Figure S3B). As no effective antibodies against A1 were available, the effect of the siRNA was evaluated using transfected recombinant A1.

To ensure that the effects of the siRNAs on mitotic cell death were not due to a general increase in apoptosis, we first analyzed the effects of the siRNAs on unperturbed cell cycle. Transfection of the siRNAs against BCL-2, BCL-XL, BCL-W, MCL1, and A1 did not result in a general increase in cell death in HCT116 (Supplementary Figure S4A) or HeLa (Supplementary Figure S4C). The duration of mitosis was also unaffected by the siRNAs in the two cell lines (Supplementary Figure S4B, D).

**Figure 3:**
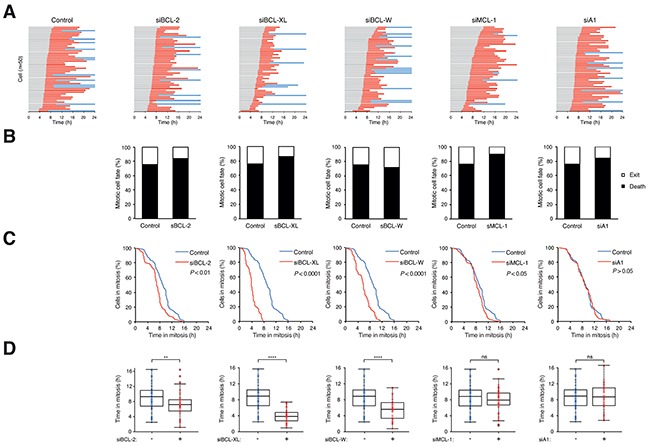
Depletion of several BCL-2-like proteins accelerates mitotic cell death **A.** Depletion of BCL-2, BCL-XL, or BCL-W accelerates PTX-mediated mitotic cell death. HeLa cells expressing histone H2B-GFP were transfected with different siRNAs. The cells were synchronized, exposed to PTX (31.3 ng/ml) and analyzed with live-cell imaging (*n*=50). Key: same as Figure 1C. **B.** Percentage of different mitotic fates from (A). **C.** The durations of mitotic block of cells from (A) are plotted using Kaplan-Meier estimator. **D.** Box-and-whisker plots showing elapsed time from mitotic entry to either mitotic exit or death from (A). Student's t-test **P <0.01; ****P<0.0001; ns *P*>0.05.

After ensuring the siRNAs could reduce the expression of anti-apoptotic BCL-2-like proteins and did not affect unperturbed mitosis, we next examined their effects on mitotic cell death. For HeLa cells, the transfection of the siRNAs did not significantly alter the overall mitotic cell fate after PTX treatment – mitotic cell death remained at around 80% (Figure 3A, B). Nevertheless, depletion of BCL-2, BCL-XL, and BCL-W significantly shortened the time before apoptosis occurred, indicating that these proteins played a role in suppressing apoptosis during the mitotic block (Figure 3C, D).

For HCT116, which underwent both mitotic cell death and slippage, the situation was more complicated because knockdown of BCL-2-like proteins resulted in the switching of mitotic cell fate in some cases. Depletion of BCL-XL switched the fate of mitosis from slippage to mitotic cell death (Supplementary Figure S5A, B), and at the same time shortened the time in mitosis (Supplementary Figure S5C, D). Knockdown of other family members (BCL-2, BCL-W, and MCL-1) also shortened the mitotic block but did not alter the cell fate significantly.

Taken together, these results provide evidence of the contribution of several anti-apoptotic BCL-2 family members including BCL-2, BCL-XL, BCL-W, and MCL-1 to the timing of mitotic cell death.

### BCL-W is a novel regulator of mitotic cell death

Several of the anti-apoptotic BCL-2 proteins identified above have been previously implicated in mitotic cell death, affirming the efficacy of the assay. These include BCL-2 [10–13], BCL-XL [15, 16], and MCL-1 [17, 18]. In contrast, the role of BCL-W in mitotic cell death has not been previously recognized.

We first ensured that the promotion of mitotic cell death by siBCL-W was not limited to PTX treatment. Cells transfected with siBCL-W were incubated with nocodazole (NOC) and analyzed with live-cell imaging as before. Supplementary Figure S6 shows that NOC-induced mitotic cell death was also significantly accelerated after depletion of BCL-W.

**Figure 4:**
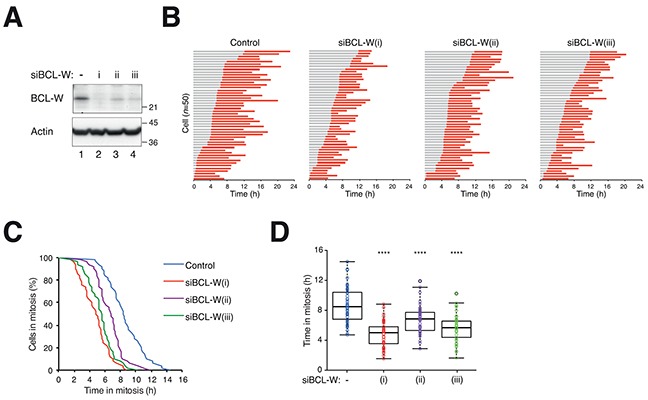
Depletion of BCL-W with multiple siRNAs accelerates mitotic cell death **A.** Depletion of BCL-W with multiple siRNAs. HeLa cells expressing histone H2B-GFP were transfected with three different siBCL-W. After 24 h, lysates were prepared and the expression of BCL-W was analyzed with immunoblotting. The positions of molecular size standards (in kDa) are indicated on the right. **B.** Depletion of BCL-W accelerates PTX-mediated mitotic cell death. HeLa cells expressing histone H2B-GFP were transfected with three different siBCL-W. The cells were synchronized, exposed to PTX (31.3 ng/ml) and analyzed with live-cell imaging (*n*=50). Key: same as Figure 1C. **C.** The durations of mitotic block of cells from (B) are plotted using Kaplan-Meier estimator. **D.** Box-and-whisker plots showing elapsed time from mitotic entry to either mitotic exit or death from (B). One-way ANOVA ****P<0.0001 (all compare to DMSO control).

We further verified that downregulation of BCL-W accelerated mitotic cell death by using two additional siRNAs. Although the other two siRNAs were noticeably less effective than the original one in depleting BCL-W (Figure 4A), they were also able to accelerate mitotic cell death (Figure 4B, C, D). These results confirmed that the effects of siBCL-W were likely to be specific.

**Figure 5:**
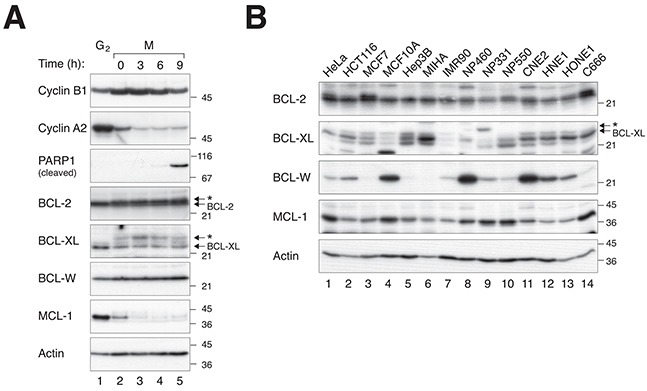
The expression of BCL-W does not change during mitotic block but varies widely among different cell lines **A.** BCL-W does not vary during mitotic block. HeLa cells were synchronized in G_2_ (using a double thymidine procedure) or further blocked in mitosis with NOC for 4 h before harvested at the indicated time points. Lysates were prepared and analyzed with immunoblotting. The accumulation of cyclin B1 and destruction of cyclin A2 confirm the cells were trapped in mitosis. Mitotic cell death was corresponded with an increase in cleaved PARP1. The asterisks indicate the phosphorylated forms of BCL-2 and BCL-XL. **B.** BCL-W expression varies widely among different cell lines. The expression of BCL-2, BCL-XL, BCL-W, and MCL-1 was detected in different cell lines. Both normal or non-transformed immortalized cells (MCF10A, MIHA, IMR90, NP460, NP331, and NP550) and cancer cell lines (the rest) were compared.

Given the importance of BCL-W in mitotic cell death, we next examined the expression of BCL-W during microtubule inhibitor-mediated mitotic blocks. Figure 5A shows that BCL-W was expressed at a relatively constant level between G_2_ and different time after incubation with NOC. This contrasted to MCL-1, which was degraded during the mitotic block. Also unlike BCL-2 and BCL-XL (which changed gel mobility during mitosis due to phosphorylation) there is no evidence that the mobility of BCL-W was altered during mitosis. Interestingly, the expression of BCL-W varied widely between different cell lines (Figure 5B). Hence the available evidence indicates that endogenous BCL-W does not vary during mitotic block.

**Figure 6:**
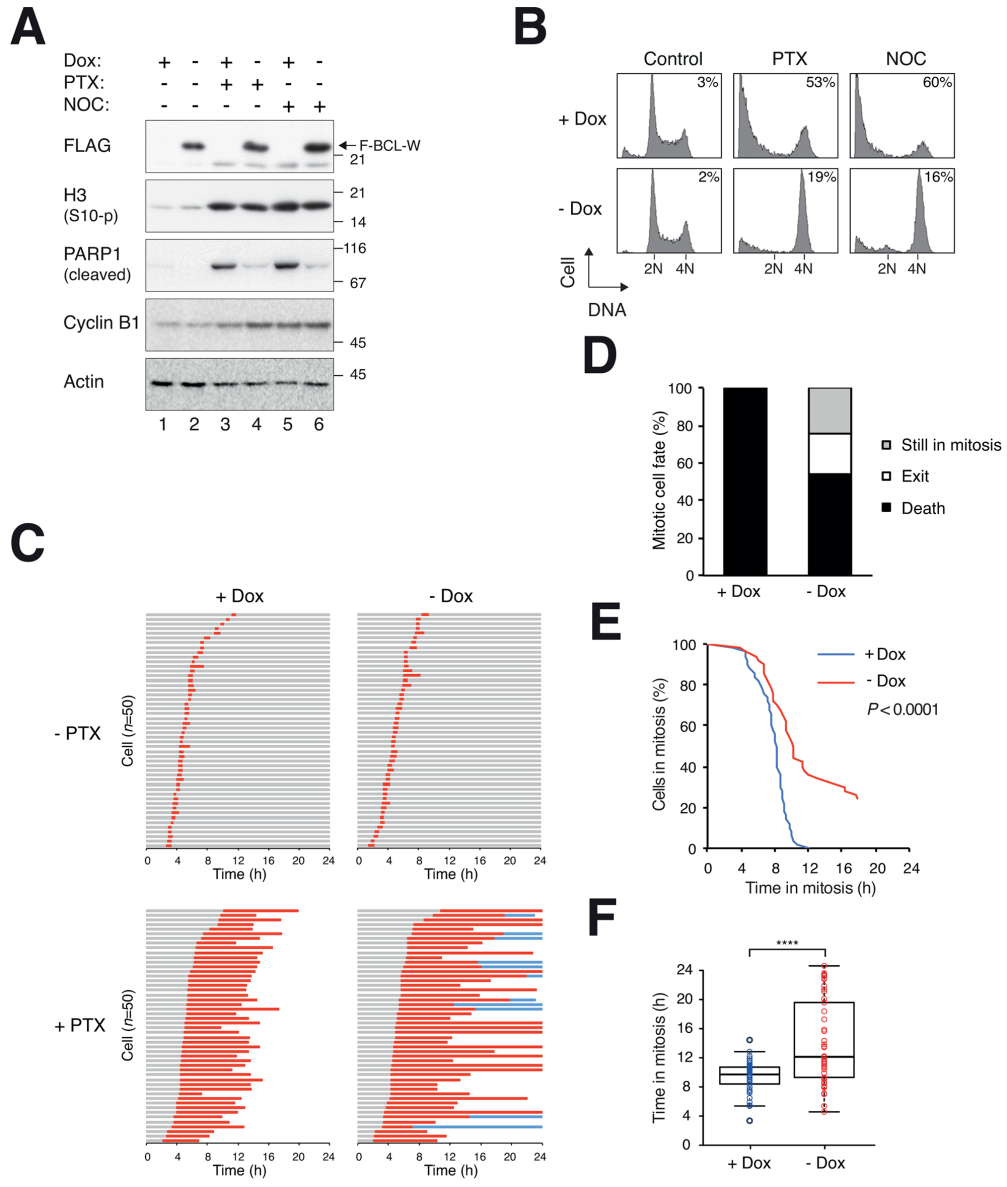
Delay of mitotic cell death by ectopic expression of BCL-W **A.** Overexpression of BCL-W inhibits PARP1 cleavage. HeLa cells expressing FLAG-BCL-W were grown in the presence or absence of doxycycline (Dox) for 3 days to turn off or on FLAG-BCL-W respectively. The cells were exposed to PTX (31.3 ng/ml) or NOC (25 ng/ml). After 24 h, the cells were harvested and analyzed with immunoblotting. **B.** Overexpression of BCL-W inhibits apoptosis. Cells were treated as in (A). After 24 h, the cells were harvested and analyzed with flow cytometry. The percentages of sub-G_1_ cells are quantified. **C.** Overexpression of BCL-W delays mitotic cell death. HeLa cells expressing FLAG-BCL-W (and also histone H2B-GFP) were grown in the presence or absence of Dox for 3 days to turn off or on FLAG-BCL-W respectively. The cells were synchronized, exposed to PTX (31.3 ng/ml) and analyzed with live-cell imaging (*n*=50). Key: same as Figure 1C. **D.** Percentage of different mitotic fates from (C). **E.** The durations of mitotic block of PTX-treated cells from (C) are plotted using Kaplan-Meier estimator. **F.** Box-and-whisker plots showing elapsed time from mitotic entry to either mitotic exit or death (cell still in mitosis at the end imaging were included). Student's t-test ****P<0.0001.

We next conducted converse experiments to see if mitotic cell death could be suppressed by overexpressed BCL-W. A cell line producing FLAG-tagged BCL-W under the control of doxycycline (Dox) was treated with PTX or NOC to block cells in mitosis (indicated by histone H3^Ser10^ phosphorylation) (Figure 6A). The associated apoptosis (indicated by PARP1 cleavage) could be suppressed by the recombinant BCL-W. This experiment also confirmed that similar to the endogenous BCL-W, mitotic blocks did not induce a change in the level or gel mobility of FLAG-BCL-W. Inhibition of mitotic apoptosis by BCL-W was further confirmed with flow cytometry (Figure 6B). Live-cell imaging revealed that although ectopic expression of BCL-W did not affect the unperturbed cell cycle, it delayed the mitotic cell death induced by PTX/NOC (Figure 6C, E, F). The delay in mitotic cell death also promoted the switch of cell fate in a population from cell death to mitotic exit (and a population of cells was still in mitosis at the end of imaging) (Figure 6D).

For comparison, we also generated cell lines overexpressing other anti-apoptotic BCL-2 family: BCL-2 (Supplementary Figure S7), BCL-XL (Supplementary Figure S8), MCL-1 (Supplementary Figure S9), and A1 (Supplementary Figure S10). These anti-apoptotic proteins also slowed down mitotic cell death similarly as BCL-W. It is notable that in contrast to the constant expression of BCL-W, the other BCL-2-like proteins were either modified (BCL-2 and BCL-XL) or degraded (MCL-1 and A1) during mitosis.

Taken together, these data indicate that although BCL-W expression remains constant during mitotic block, it appears to contribute to setting a threshold of anti-apoptotic activity during mitosis.

### Ablation of *BCL-W* gene promotes mitotic cell death

To address unequivocally the involvement of BCL-W in mitotic cell death, its gene was disrupted using CRISPR-Cas9 gene editing tools. Genomic DNA sequencing validated that deletions occurred at the CRISPR-targeting region of *BCL-W*, resulting in premature termination of the gene products (Supplementary Figure S11).

**Figure 7:**
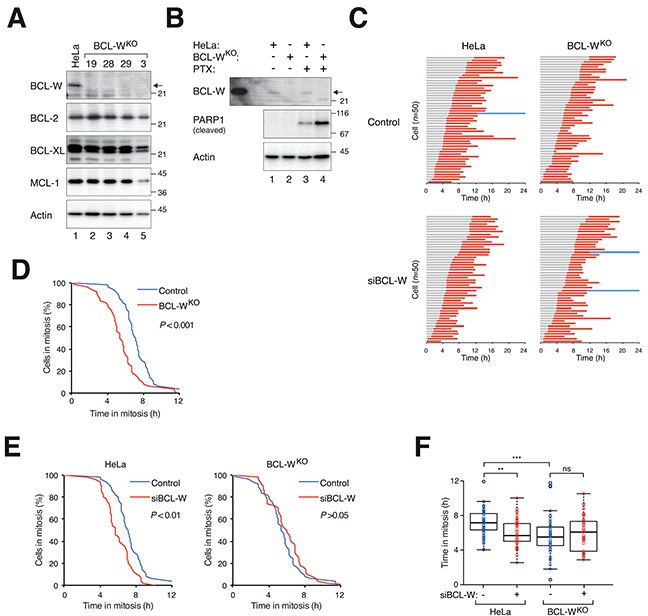
Disruption of *BCL-W* gene accelerates mitotic cell death **A.** Disruption of BCL-W with CRISPR-Cas9. HeLa cells expressing histone H2B-GFP were transfected with a CRISPR-Cas9 construct targeting BCL-W and individual colonies were isolated. Lysates were prepared and the expression of BCL-W and other BCL-2-related proteins was detected with immunoblotting. HeLa cell lysates were loaded in lane 1. Clone #28 was used in the rest of this study. **B.** Knockout of BCL-W sensitizes cells to PTX. HeLa or BCL-W-knockout (BCL-W^KO^) cells were incubated with either DMSO or PTX (31.3 ng/ml) for 24 h. Lysates were produced and the expression of cleaved PARP1 was detected with immunoblotting. HeLa cells overexpressing FLAG-BCL-W were loaded on the left a marker. **C.** Knockout of BCL-W accelerates PTX-mediated mitotic cell death. HeLa or BCL-W^KO^ cells expressing histone H2B-GFP transfected with either control or siBCL-W. The cells were synchronized, exposed to PTX (31.3 ng/ml) and analyzed with live-cell imaging (*n*=50). Key: same as Figure 1C. **D.** The durations of mitotic block of PTX-treated HeLa versus BCL-W^KO^ cells from (C) are plotted using Kaplan-Meier estimator. **E.** BCL-W^KO^ cells are insensitive to the effects of siBCL-W on mitotic cell death. The durations of mitotic block of PTX-treated HeLa (left) or BCL-W^KO^ (right) in the presence or absence of siBCL-W from (C) are plotted using Kaplan-Meier estimator. **F.** Box-and-whisker plots showing elapsed time from mitotic entry to either mitotic exit or death from (C). Student's t-test **P<0.01; ***P<0.001; ns *P*>0.05.

Knockout of BCL-W in HeLa cells did not affect the expression of other anti-apoptotic BCL-2-related proteins examined (Figure 7A). The loss the BCL-W increased the apoptosis induced by PTX (as detected by PARP1 cleavage), supporting a role of BCL-W in antagonizing mitotic cell death (Figure 7B). This was further illustrated by the shortening of mitotic block before cell death occurred (Figure 7C, D, F). The ability of siBCL-W in speeding up mitotic cell death in HeLa cells was abolished in BCL-W-deficient cells (Figure 7E, F), further validating that the effects of the siBCL-W was specific for BCL-W.

Collectively, these data show that BCL-W controls the timing of microtubule inhibitor-mediated apoptosis during mitosis.

## DISCUSSION

A controversial issue in the field is whether the ability to promote of mitotic cell death alone correlates with the effectiveness of antimitotic drugs. Although mitotic slippage prevents immediate cell death, the G_1_ cells generated are less fit to propagate than normal cells, in part because S phase entry after mitotic slippage is prevented by a p53-dependent mechanism [19]. This p53-dependent arrest is mainly caused by DNA damage or centrosomal stress during the aberrant mitosis rather than tetraploidization per se [20]. In p53-defective cells, genome reduplication and multipolar mitosis become unchecked after mitotic slippage, thereby promoting further chromosomal instability. Hence tipping the balance towards mitotic cell death is likely to improve the usefulness of antimitotic drug therapies.

Inhibition of apoptosis with a caspase inhibitor extended PTX-treated mitosis by at least 45% (Figure 2). The accumulation of apoptotic signals during mitosis can be contributed by both an increase of pro-apoptotic molecules and/or a decrease of anti-apoptotic molecules. At the same time, other pro- and anti-apoptotic molecules expressed at constant levels may determine when the threshold of apoptosis is breached. The number of BCL-2-like proteins adds to the complication of identifying which ones are critical in controlling mitotic cell death. As overexpression of all anti-apoptotic BCL-2 proteins could delay mitotic cell death (Figures 6, Supplementary Figure S7-S10), loss-of-function studies are required to reveal the importance of each protein in different cell types.

Knockdown of BCL-W with siRNA significantly accelerated mitotic cell death after treatment with PTX (Figure 4) or NOC (Supplementary Figure S6) in both HeLa and HCT116 cells. The average time that cells were blocked in mitosis before apoptosis occurred was about halved. Acceleration of mitotic cell death could also be recapitulated using BCL-W^KO^ cells (Figure 7F). Unperturbed mitosis was not affected by knockdown of BCL-W with siRNA (Supplementary Figure S4) or in BCL-W^KO^ cells (data not shown). The effects of the knockdown of BCL-W was likely to be specific because (i) the acceleration of mitotic cell death could be reproduced using several independent siRNAs (Figure 4); (ii) the siRNA was no longer able to promote mitotic cell death in a BCL-W-deficient background (Figure 7). The model of how the mitotic cell fate is determined is based on the competition between the rate of mitotic slippage versus mitotic cell death (see Introduction). This model assumes that mitotic slippage and mitotic cell death are independently regulated. Thus increasing BCL-W delayed cell death because the cell death threshold was increased (Figure 6). Conversely, decreasing (Figure 4) or removing (Figure 7) BCL-W accelerated cell death because the death threshold was lowered.

Unlike MCL-1 and A1, the expression of BCL-W was unchanged during mitotic block (Figure 5A). It is generally believed that anti-apoptotic proteins varying during mitotic block may play a key role in regulating mitotic cell death. However, it can also be argued that proteins that are degraded probably do not have a strong influence on the timing of mitotic cell death because they are eliminated rapidly irrespective of the starting level. For example, we found that the degradation of MCL-1 occurred relatively early during mitotic block and was temporally distinct from mitotic cell death (Figure 5A). In agreement with this, knockdown of MCL-1 exerted no or marginal effect on the timing of mitotic cell death in HeLa and HCT116 respectively (Figures 3 and Supplementary Figure S5). The exception is under conditions when these proteins were overexpression, which prevented their complete degradation and delayed mitotic cell death (Supplementary Figure S9).

BCL-2 and BCL-XL are phosphorylated by cyclin B–CDK1 during mitosis (see Introduction), resulting in gel mobility shifts. In contrast, gel mobility shifts of BCL-W were not observed during mitosis (Figure 5A). Although BCL-W was expressed at a constant level during mitotic block, it was expressed at varying levels in different cell lines (Figure 5B). One model is that together with other BCL-2-like proteins, BCL-W contributes to setting a threshold for mitotic cell death in different cells.

An obvious clinical implication of this study is that in addition to proteins including BCL-2 and BCL-XL, the expression of BCL-W is important for antimitotic drug-mediated cytotoxicity. Another clinical-relevant aspect is that several chemicals in clinical development are inhibitors of BCL-2, BCL-XL, and BCL-W. These include ABT-263 (navitoclax) [21] and ABT-737 [22]. The relative expression of BCL-W in normal and cancer cells is expected to affect the effectiveness of these chemicals either alone or in combination with antimitotic drugs.

## MATERIALS AND METHODS

### DNA constructs

Human BCL-W (IMAGE ID 8143792), BCL-XL (Addgene, ID8749), MCL-1 (Addgene, ID21605), and A1 (IMAGE ID 3920808) were amplified with PCR using the following pairs of oligonucleotides: 5′-CCCCATGGCGACCCCAGCCTC-3′ and 5′-CTGAATTCTCACTTGCTAGCAAAAAAG-3′ (BCL-W); 5′-TTGA ATTCATGTCTCAGAGCAA-3′ and 5′-GTGAATTCTCATT TCCGACTGA-3′ (BCL-XL); 5′-GAGAATTCATGTTTG GCCTCAAAAGAAACGCGGTA-3′ and 5′-CAGGATCCCTATCTTATTAGATATGCCAAACCAGC-3′ (MCL-1); 5′-CAGGGAATTCATGACAGACTGTG-3′ and 5′-TCCTGGATCCTCAACAGTATTGCTTC-3′ (A1). The PCR products were cut with *Nco* I-*Eco*R I (BCL-W), *Eco*R I (BCL-XL), or *Bam*H I-*Eco*R I (MCL-1 and A1), and ligated into a modified pUHD-P3 [23] lacking one *Xho* I site to obtain FLAG-tagged expression constructs. BCL-2 in pCMV-SPORT6 (Image ID:4511027) was obtained from Source Biooscience (Nottingham, UK). The BCL-2 cDNA was amplified with PCR using the primers 5′-AACCATGGCGCACGCTGGGAGAA-3′ and 5′-TGGAATTCTCACTTGTGGCCCAGATA-3′; the PCR product was cut with *Nco* I–*Eco*R I and ligated into pUHD-P3 [23]. The *Xho* I–*Eco*R I fragment of this construct was ligated into pUHD-P3T(PUR) [23] to obtain FLAG-BCL-2 in pUHD-P3T(PUR). To generate the GST-tagged BCL-XL for bacterial expression, BCL-XL in pSFFV-neo (Addgene, ID 8749) was amplified with PCR using oligonucleotides 5′-TTGAATTCAAATGTCTCAGAGCAA-3′ and 5′-GTGAA TTCTCATTTCCGACTGA-3′; the product was cut with *Eco*R I and ligated into pGEX-KG [24]. CRISPR-Cas9(BCL-W) construct targeting ACTTTGTAGGTTATAAGCTG was obtained from Horizon (Cambridge, UK).

### *BCL-W* gene disruption

Histone H2B-GFP-expressing HeLa cells were first transfected with the CRISPR-Cas9(BCL-W) plasmid together with a plasmid expressing a puromycin-resistant gene. Transfected cells were selected transiently in puromycin-containing medium for 2 days followed by growing in medium without puromycin for 3 days. The cells were then seeded onto 96-well plates with limiting dilution to obtain single cell-derived colonies. The colonies were then analyzed with immunoblotting to confirm successful gene disruption.

### Genomic DNA sequence analysis

Genomic DNA from 1×10^7^ cells was prepared as described [25]. Fragment close to the CRISPR-targeting sites were amplified with PCR using 5′-GCAGCGGCCTGACCCGTGAGATCCCTAAC-3′ and 5′-AGCATCCTTTGCCAAGGCTTGCCTGACCACC-3′. The genomic PCR products were then sequenced with 5′-AGCATCCTTTGCCAAGGCTTGCCTGACCACC-3′ and the heterozygous deletions were resolved with CodonCode Aligner (CodonCode Corporation, Centerville, MA, USA).

### Quantitative real-time PCR

The conditions of RT-PCR were as previously described [26] using the following primers 5′-AAGATT GATGGGATCGTTGC-3′ and 5′-GCGGAACACTTGATTC TGGT-3′ (BCL-2); 5′-TCTGGTCCCTTGCAGCTAGT-3′ and 5′-CAGGGAGGCTAAGGGGTAAG-3′ (BCL-XL); 5′-GGCGCACCTTCTCTGATCTG-3′ and 5′-GTGGTTC CATCTCCTTGTTGACA-3′ (BCL-W); 5′-CTTCCAAGGA TGGGTTTGTG-3′ and 5′-GCTAGGTTGCTAGGGTGCA A-3′ (MCL-1); 5′-TACAGGCTGGCTCAGGACTATCT-3′ and 5′-TCCATCACTTGGTTGAATAGTGTTC-3′ (A1); 5′-GGGAAATCGTGCGTGACATT-3′ and 5′-GGAACCG CTCATTGCCAAT-3′ (actin).

### Cell culture

The HeLa used in this study was a clone expressing the tTA tetracycline transactivator [27]. HCT116 (colorectal carcinoma) was a gift from Bert Vogelstein (The Johns Hopkins University). Normal human fibroblasts (IMR90) and breast cancer cell lines (MCF7 and MCF10A) were obtained from American Type Culture Collection (Manassas, VA, USA). Nasopharyngeal carcinoma cell lines (C666-1, CNE2, HNE1, and HONE1) and telomerase-immortalized nasopharyngeal epithelial cell lines (NP361, NP460, and NP550) were as previously described [28]. Normal liver (MIHA) and hepatocellular carcinoma cell lines (Hep3B) were as previously described [29].

HeLa [30] and HCT116 [31] cells stably expressing histone H2B-GFP were used in live-cell imaging. To generate cells stably expressing FLAG-tagged anti-apoptotic BCL-2 family, histone H2B-GFP-expressing HeLa cells were transfected with FLAG-BLC-2, BCL-XL, BCL-W, MCL-1, or A1 in pUHD-P3 respectively followed by selection with puromycin. The cells were grown in the presence of Dox to suppress the expression of the BCL-2-like proteins. After two weeks of selection, single cell-derived colonies were isolated. Cells were propagated in Dulbecco's modified Eagle's medium (DMEM) supplemented with 10%(v/v) calf serum (HeLa) or fetal bovine serum (HCT116) and 50 U/ml penicillin-streptomycin (Life Technologies, Carlsbad, CA, USA) in a humidified incubator at 37°C in 5% CO_2_. Cells were transfected with plasmids using a calcium phosphate precipitation method [25]. Cell-free extracts were prepared as described previously [32]. Cell cycle synchronization was performed as described [33]. Unless stated specifically, cells were treated with the following reagents at the indicated final concentration: chloroquine diphosphate (Santa Cruz; 10 μM); doxycycline hydrochloride (Sigma-Aldrich, St. Louis, MO, USA; 2 μg/ml), necrostatin (Selleckchem; 10 μM), nocodazole (Sigma-Aldrich; 0.1 μg/ml), paclitaxel (USB, Cleveland, OH, USA; 500 ng/ml), puromycin (Sigma-Aldrich; 30 μg/ml), and Z-VAD-FMK (pan-caspase inhibitor) (Enzo Life Sciences, Farmingdale, NY, USA; 20 μM).

### RNA interference

siRNAs targeting BCL-2 (GGAUGACUGAGUACCUGAATT, siBCL-2), BCL-W (GCAGACUUUGUAGGUUAUATT, siBCL-W), BCL-XL (GCCAUCAAUGGCAACCCAUTT, siBCL-XL), MCL-1 (GCCUUCCAAGGAUGGGUUUTT, siMCL-1) were manufactured by RiboBio (Guangzhou, China). Other siRNAs targeting A1 (AAGGAGUUUGAAGACGGCAUCTT, siA1) and BCL-W (CCUGAGCACUGAUCACCUUTT, siBCL-W#2; CCGCCUUGUAGCCUUCUUUTT, siBCL-W#3) were manufactured by Genepharma (Shanghai, China). Cells were transfected with siRNAs (15 nM) using Lipofectamine™ RNAiMAX (Life Technologies).

### Antibodies and immunological methods

Monoclonal antibodies against β-actin, cyclin A2 [34], FLAG (Sigma-Aldrich), BCL-2 (DAKO, Glostrup, Denmark), cleaved PARP1 (BD Biosciences, Franklin Lakes, NJ, USA) were obtained from the indicated suppliers or described previously. Polyclonal antibodies against phosphor-histone H3^Ser10^, MCL-1 (Santa Cruz Biotechnology, Santa Cruz, CA, USA), and BCL-W (Cell Signaling Technology, Beverly, MA, USA) were obtained from the indicated suppliers. Antibodies against cyclin B1 were gifts from Julian Gannon (Cancer Research UK). Rabbit polyclonal antibody against BCL-XL was raised against bacterially expressed GST-BCL-XL. Immunoblotting was performed as previously described [32].

### Live-cell imaging

The setup and conditions of time-lapse microscopy of living cells were as described previously [28].

### Flow cytometry

Flow cytometry analysis after propidium iodide staining was performed as described previously [35].

### Statistical analysis

Box-and-whisker plots (center lines show the medians; box limits indicate interquartile range; whiskers extend to the most extreme data points that were no more than 1.5 times the interquartile range from the 25^th^ and 75^th^ percentiles) were generated with BoxPlotR [36]. Student's t-test or one-way ANOVA (for box-and-whisker plots) and log-rank test (for Kaplan-Meier estimator) were used to calculate statistical significance (*P<0.05; **P<0.01; ***P<0.001; ****P<0.0001; ns *P*>0.05).

## SUPPLEMENTARY FIGURES AND VIDEOS









## Abbreviations

Dox: doxycycline
NOC: nocodazole
PTX: paclitaxel
