# Supplementary material for: BCL-W is a regulator of microtubule inhibitor-induced mitotic cell death
Source: Oncotarget. 2016 May 25;7(25):38718–30. doi: 10.18632/oncotarget.9586 (PMC5122423; doi:10.18632/oncotarget.9586)
Supplement: Supplementary file 1 [file oncotarget-07-38718-s001.pdf]

BCL-W is a regulator of microtubule inhibitor-induced mitotic cell death

SUPPLEMENTARY FIGURES AND VIDEOS

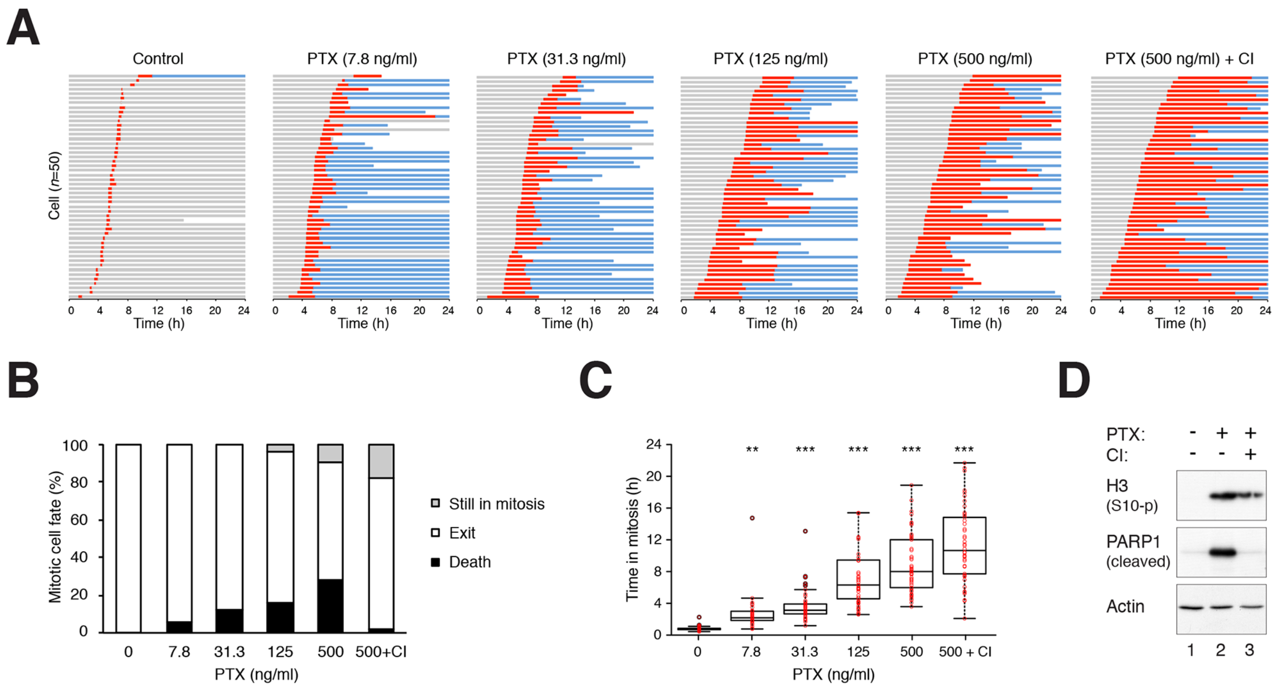

Supplementary Figure S1: PTX-induced mitotic cell death can be abolished with a pan-caspase inhibitor in HCT116 cells. A. PTX-mediated mitotic cell death can be abolished with a pan-caspase inhibitor. Live traces of HCT116 cells (expressing histone H2B-GFP) exposed to either DMSO or a 4-fold increasing concentration of PTX for 24 h (*n*=50). Cells treated with 500 ng/ml of PTX was treated with either DMSO or Z-VAD-FMK (CI). Key: same as Figure 1C. **B.** Percentage of different mitotic fates from (A). **C.** Box-and-whisker plots showing elapsed time between mitotic entry and mitotic cell death/exit (*n*=50 for each treatment). One-way ANOVA $^{**}P<0.01$; $^{***}P<0.001$ (all compare to DMSO control). **D.** Inhibition of caspases abolished PTX-mediated apoptosis. HCT116 cells were exposed to PTX (500 ng/ml) in the presence or absence of Z-VAD-FMK (CI) for 24 h. Lysates were prepared and analyzed with immunoblotting.

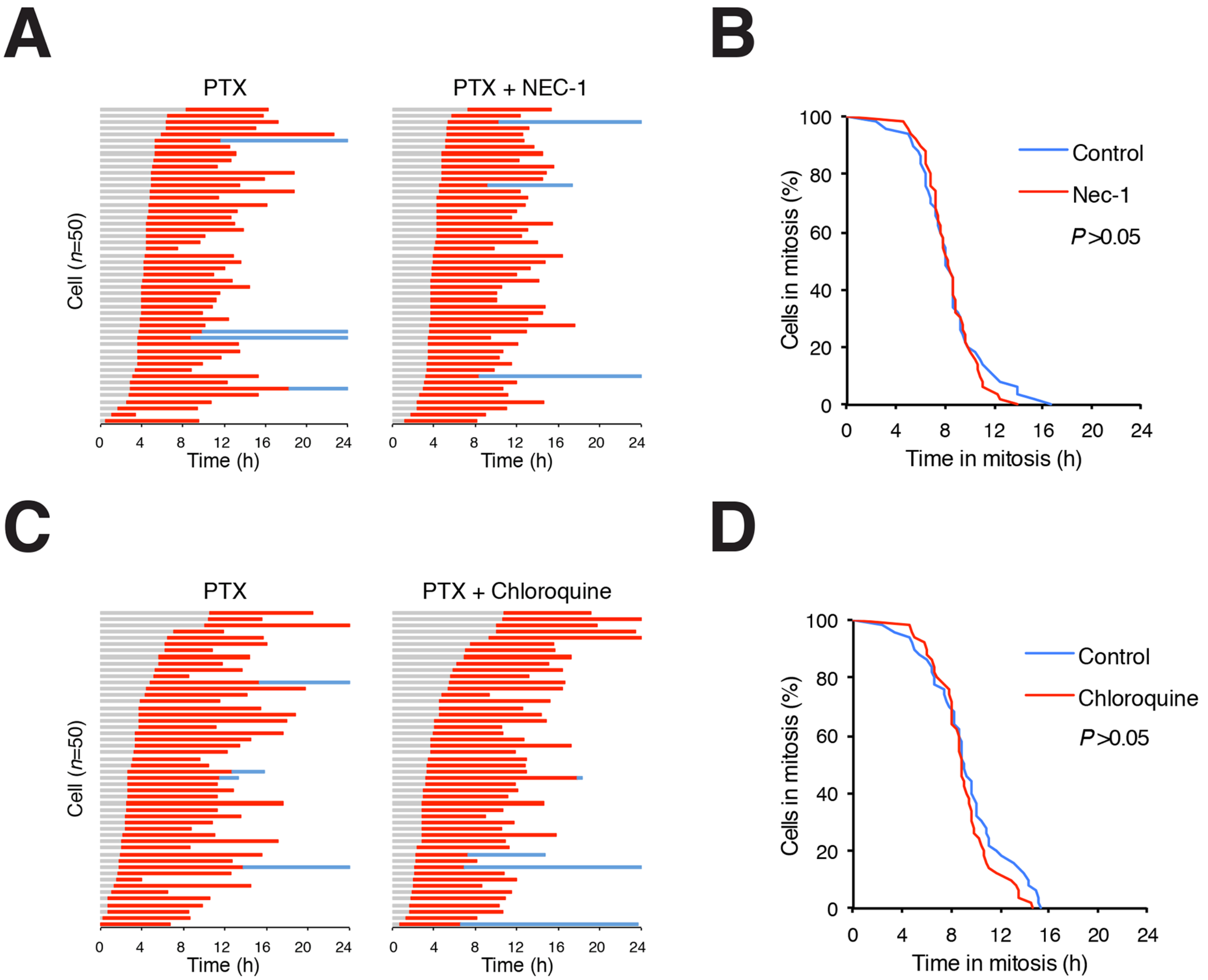

Supplementary Figure S2: Inhibition of necroptosis or autophagy does not affect mitotic cell death. A. Inhibition of necroptosis does not delay PTX-mediated mitotic cell death. Live trace of HeLa cells exposed to PTX (31.3 ng/ml) in the presence or absence of necrostatin-1 (NEC-1) (*n*=50). Key: same as Figure 1C. **B.** The durations of mitotic block of cells from (A) are plotted using Kaplan-Meier estimator. **C.** Inhibition of autophagy does not delay PTX-mediated mitotic cell death. Live trace of HeLa cells exposed to PTX (31.3 ng/ml) in the presence or absence of chloroquine (*n*=50). Key: same as Figure 1C. **D.** The durations of mitotic block of cells from (C) are plotted using Kaplan-Meier estimator.

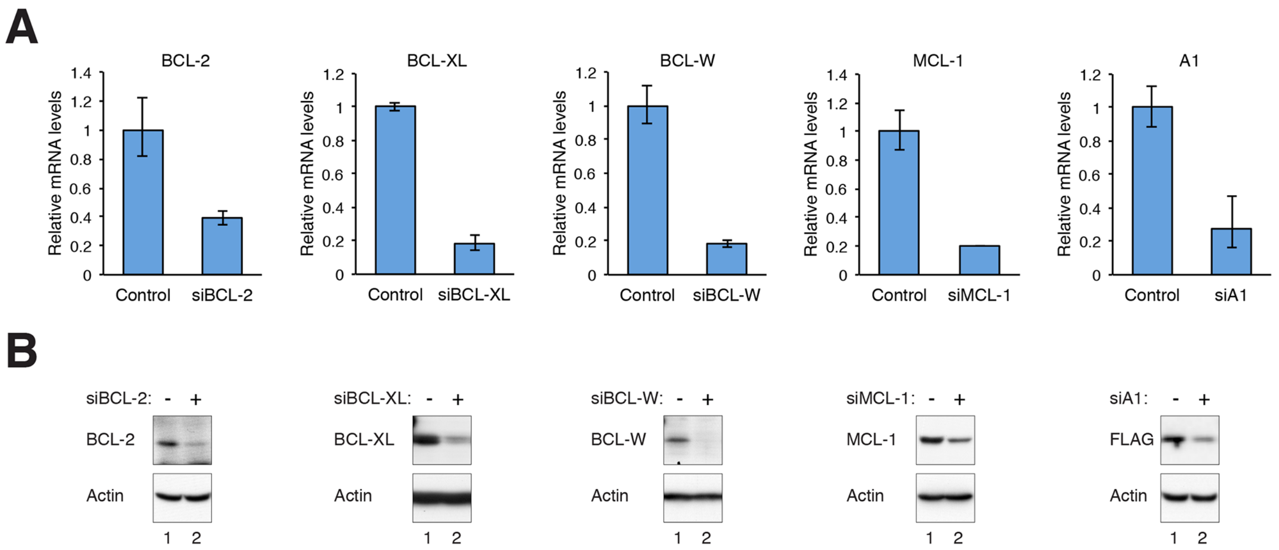

Supplementary Figure S3: Depletion of anti-apoptotic BCL-2 family with siRNAs. A. Downregulation of anti-apoptotic BCL-2 family by siRNA. HCT116 cells were transfected with siRNA targeting individual BCL-2 family member. The cells were harvested after 24 h. Total mRNA was extracted and the relative mRNA levels of the targeted BCL-2 family were determined by quantitative real-time PCR and normalized with actin. Mean±95% CI of triplicates. **B.** Protein levels of anti-apoptotic BCL-2 family after siRNA transfection. HCT116 cells were transfected with siRNA targeting individual BCL-2 family member. The cells were harvested after 24 h. Cell-free extracts were prepared and the corresponding BCL-2-like proteins were detected with immunoblotting. Uniform loading of lysates was confirmed by immunoblotting for actin. As no effective antibodies against A1 was available, FLAG-A1 was expressed by transient transfection and detected using anti-FLAG antibodies.

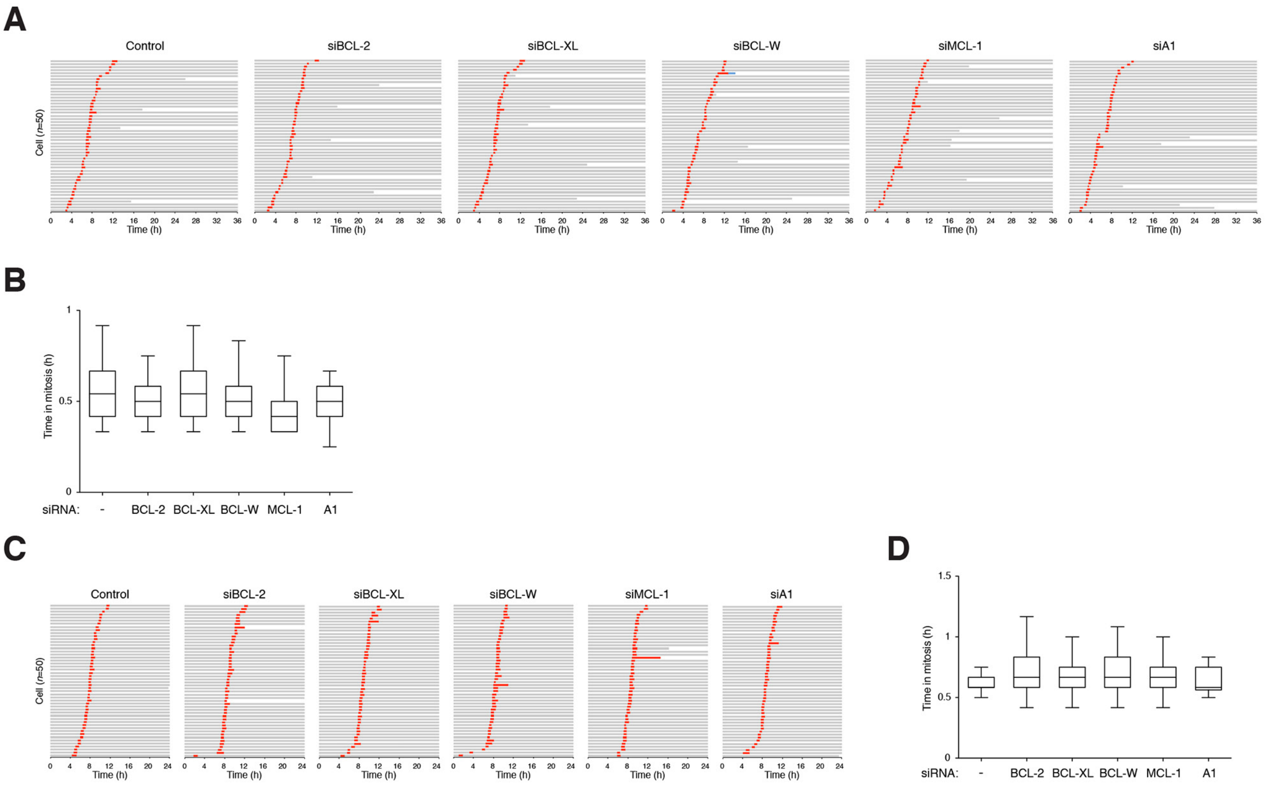

Supplementary Figure S4: Depletion of anti-apoptotic BCL-2-like proteins does not affect normal mitotic progression or cell viability. A. Live trace of HCT116 cells transfected with siRNA targeting the indicated anti-apoptotic members in the BCL-2 family (*n*=50). Key: same as Figure 1C. **B.** Box-and-whisker plots showing elapsed time between mitotic entry and exit from (A) (*n*=50 for each group). **C.** Live trace of HeLa cells transfected with siRNA targeting the indicated anti-apoptotic members in the BCL-2 family (*n*=50). Key: same as Figure 1C. **D.** Box-and-whisker plots showing elapsed time between mitotic entry and exit from (C) (*n*=50 for each group).

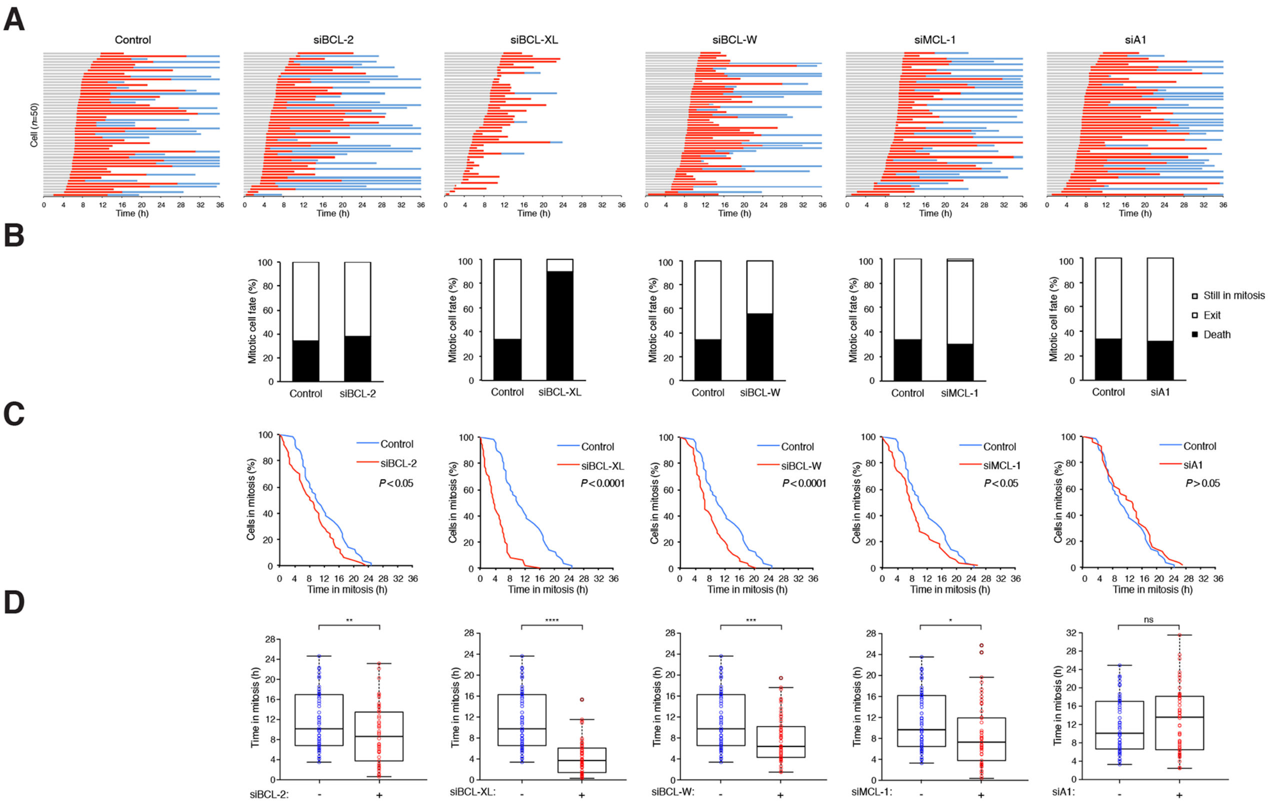

Supplementary Figure S5: Depletion of several BCL-2-like proteins curtails mitotic block in HCT116 cells. A. Depletion of BCL-2, BCL-XL, BCL-W, or MCL-1 accelerates PTX-mediated mitotic cell death or exit. HCT116 cells expressing histone H2B-GFP were transfected with different siRNAs. The cells were synchronized, exposed to PTX (500 ng/ml) and analyzed with live-cell imaging (*n*=50). Key: same as Figure 1C. **B.** Percentage of different mitotic fates from (A). **C.** The durations of mitotic block of cells from (A) are plotted using Kaplan-Meier estimator. **D.** Box-and-whisker plots showing elapsed time from mitotic entry to either mitotic exit or death. Student's t-test **P*<0.05; ***P*<0.01; ****P*<0.001; *****P*<0.0001; ns *P*>0.05.

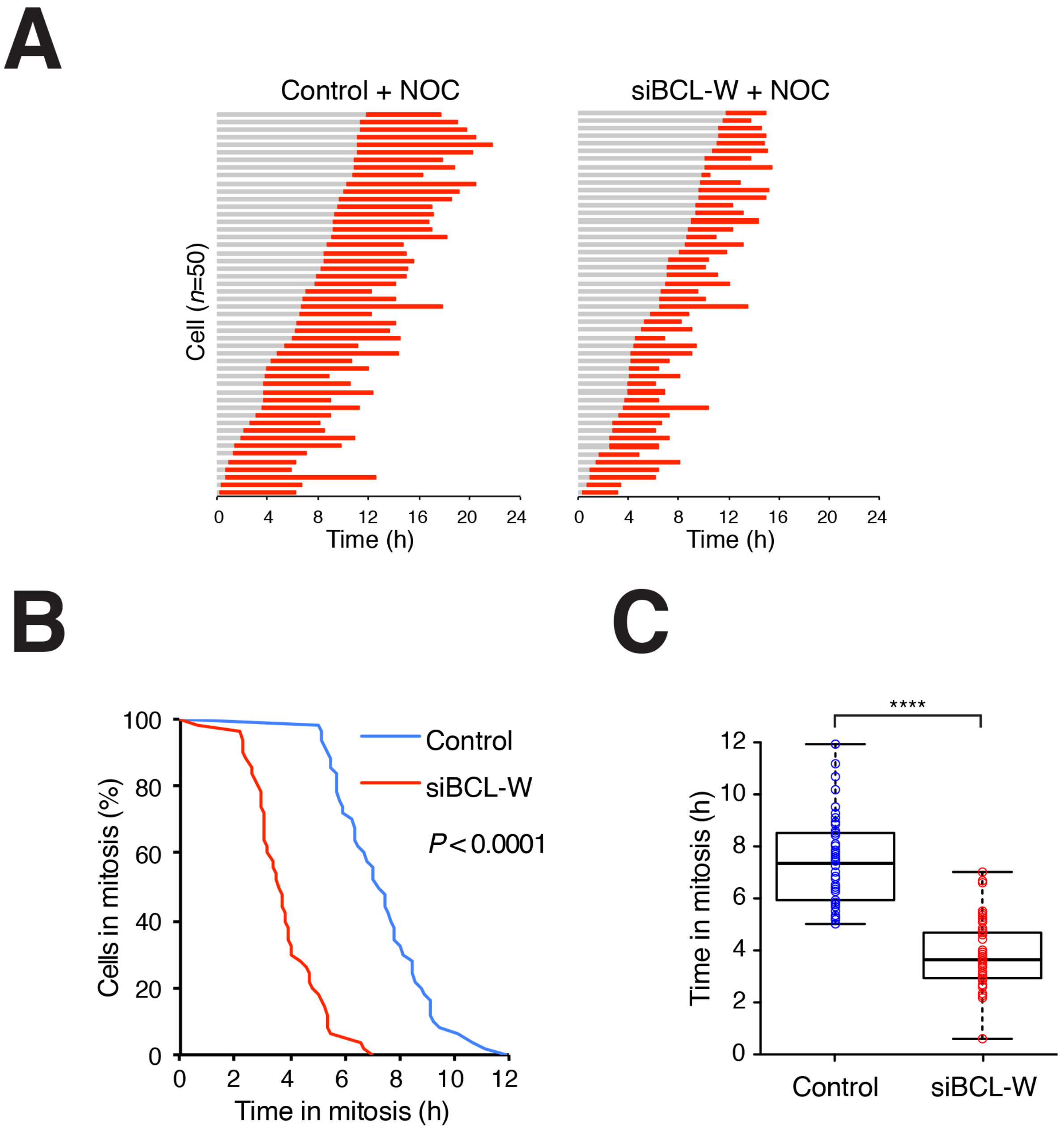

Supplementary Figure S6: Depletion of BCL-W accelerates NOC-induced mitotic cell death. A. Depletion of BCL-W accelerates NOC-induced mitotic cell death. HeLa cells expressing histone H2B-GFP were transfected with siBCL-W. The cells were synchronized, exposed to NOC (25 ng/ml) and analyzed with live-cell imaging (*n*=50). Key: same as Figure 1C. **B.** The durations of mitotic block of cells from (A) are plotted using Kaplan-Meier estimator. **C.** Box-and-whisker plots showing elapsed time from mitotic entry to mitotic death. Student's t-test ****P<0.0001.

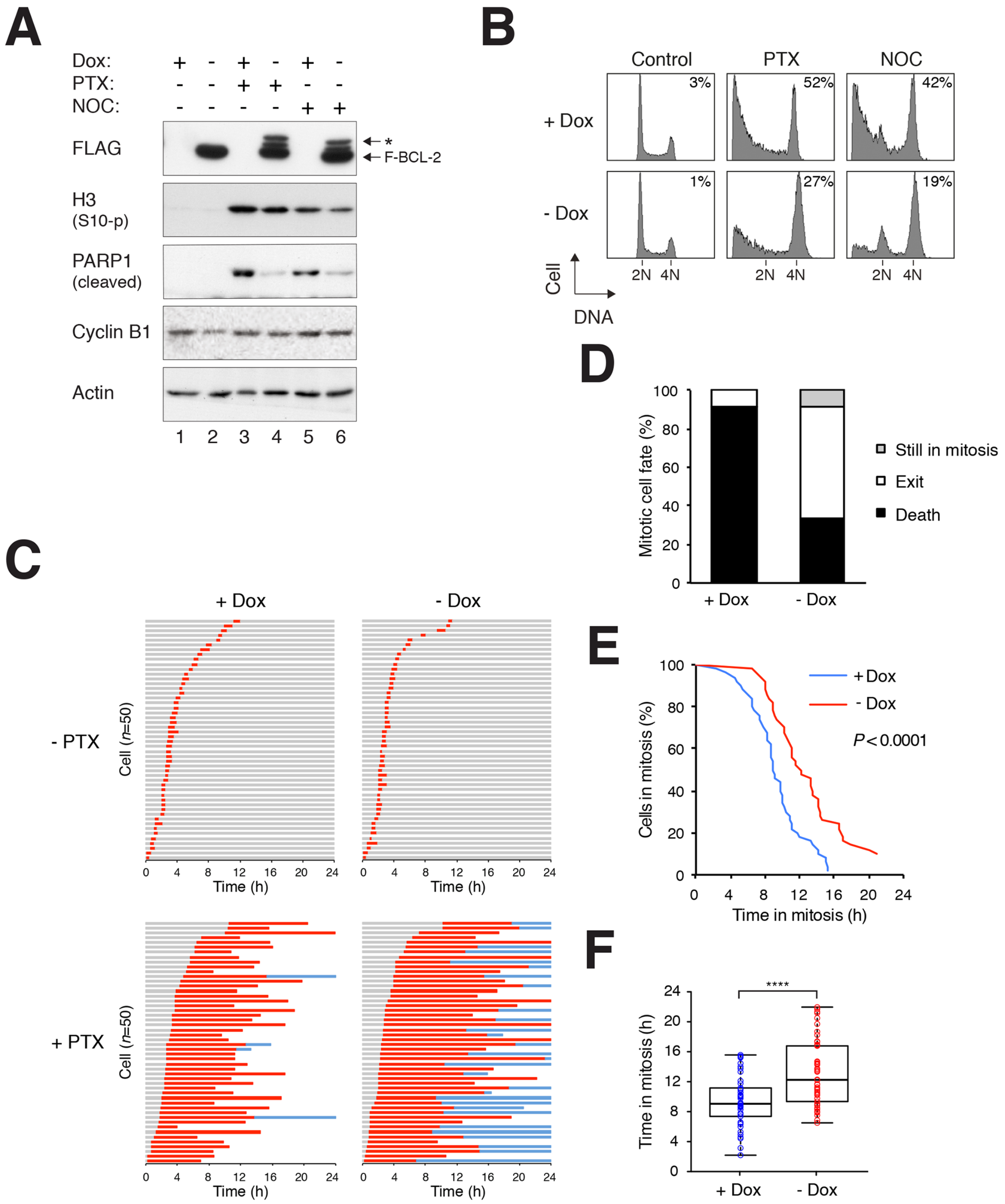

Supplementary Figure S7: Delay of mitotic cell death by ectopic expression of BCL-2. A. BCL-2 inhibits PARP1 cleavage. HeLa cells expressing FLAG-BCL-2 were grown in the presence or absence of Dox for 3 days to turn off or on FLAG-BCL-2 respectively. The cells were exposed to PTX (31.3 ng/ml) or NOC (25 ng/ml). After 24 h, the cells were harvested and analyzed with immunoblotting. The asterisk indicates the phosphorylated form of BCL-2. **B.** BCL-2 inhibits apoptosis. Cells were treated as in (A). After 24 h, the cells were harvested and analyzed with flow cytometry. The percentages of sub-G_1 cells are quantified. **C.** BCL-2 delays mitotic cell death. HeLa cells expressing FLAG-BCL-2 (and also histone H2B-GFP) were grown in the presence or absence of Dox for 3 days to turn off or on FLAG-BCL-2 respectively. The cells were synchronized, exposed to PTX (31.3 ng/ml) and analyzed with live-cell imaging (*n*=50). Key: same as Figure 1C. **D.** Percentage of different mitotic fates from (C). **E.** The durations of mitotic block of PTX-treated cells from (C) are plotted using Kaplan-Meier estimator. **F.** Box-and-whisker plots showing elapsed time from mitotic entry to either mitotic exit or death (cell still in mitosis at the end imaging were included). Student's t-test ****P<0.0001.

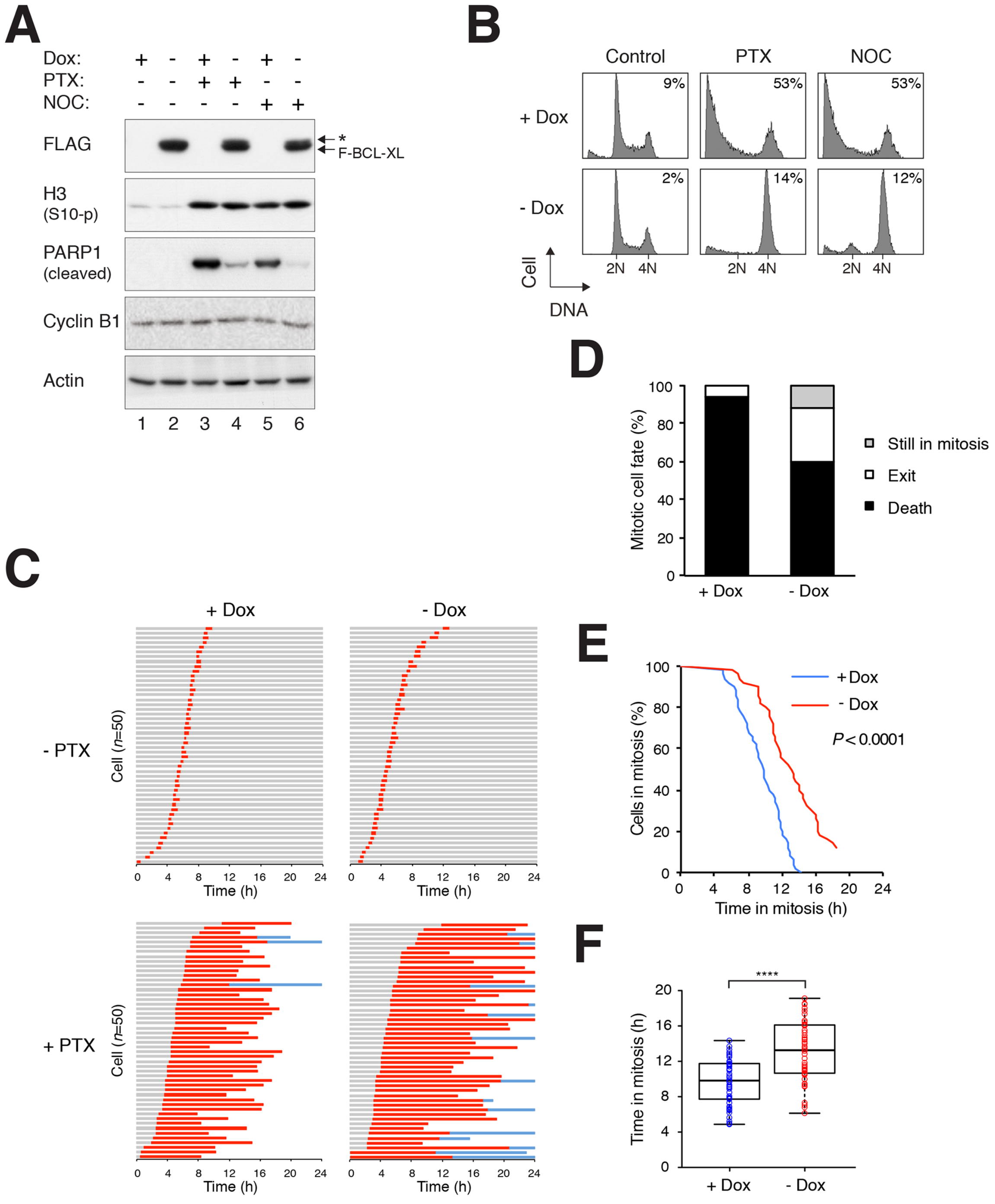

Supplementary Figure S8: Delay of mitotic cell death by ectopic expression of BCL-XL. A. BCL-XL inhibits PARP1 cleavage. HeLa cells expressing FLAG-BCL-XL were grown in the presence or absence of Dox for 3 days to turn off or on FLAG-BCL-XL respectively. The cells were exposed to PTX (31.3 ng/ml) or NOC (25 ng/ml). After 24 h, the cells were harvested and analyzed with immunoblotting. The asterisk indicates the phosphorylated form of BCL-XL. **B.** BCL-XL inhibits apoptosis. Cells were treated as in (A). After 24 h, the cells were harvested and analyzed with flow cytometry. The percentages of sub-G_1 cells are quantified. **C.** BCL-XL delays mitotic cell death. HeLa cells expressing FLAG-BCL-XL (and also histone H2B-GFP) were grown in the presence or absence of Dox for 3 days to turn off or on FLAG-BCL-XL respectively. The cells were synchronized, exposed to PTX (31.3 ng/ml) and analyzed with live-cell imaging (*n*=50). Key: same as Figure 1C. **D.** Percentage of different mitotic fates from (C). **E.** The durations of mitotic block of PTX-treated cells from (C) are plotted using Kaplan-Meier estimator. **F.** Box-and-whisker plots showing elapsed time from mitotic entry to either mitotic exit or death (cell still in mitosis at the end imaging were included). Student's t-test ****P<0.0001.

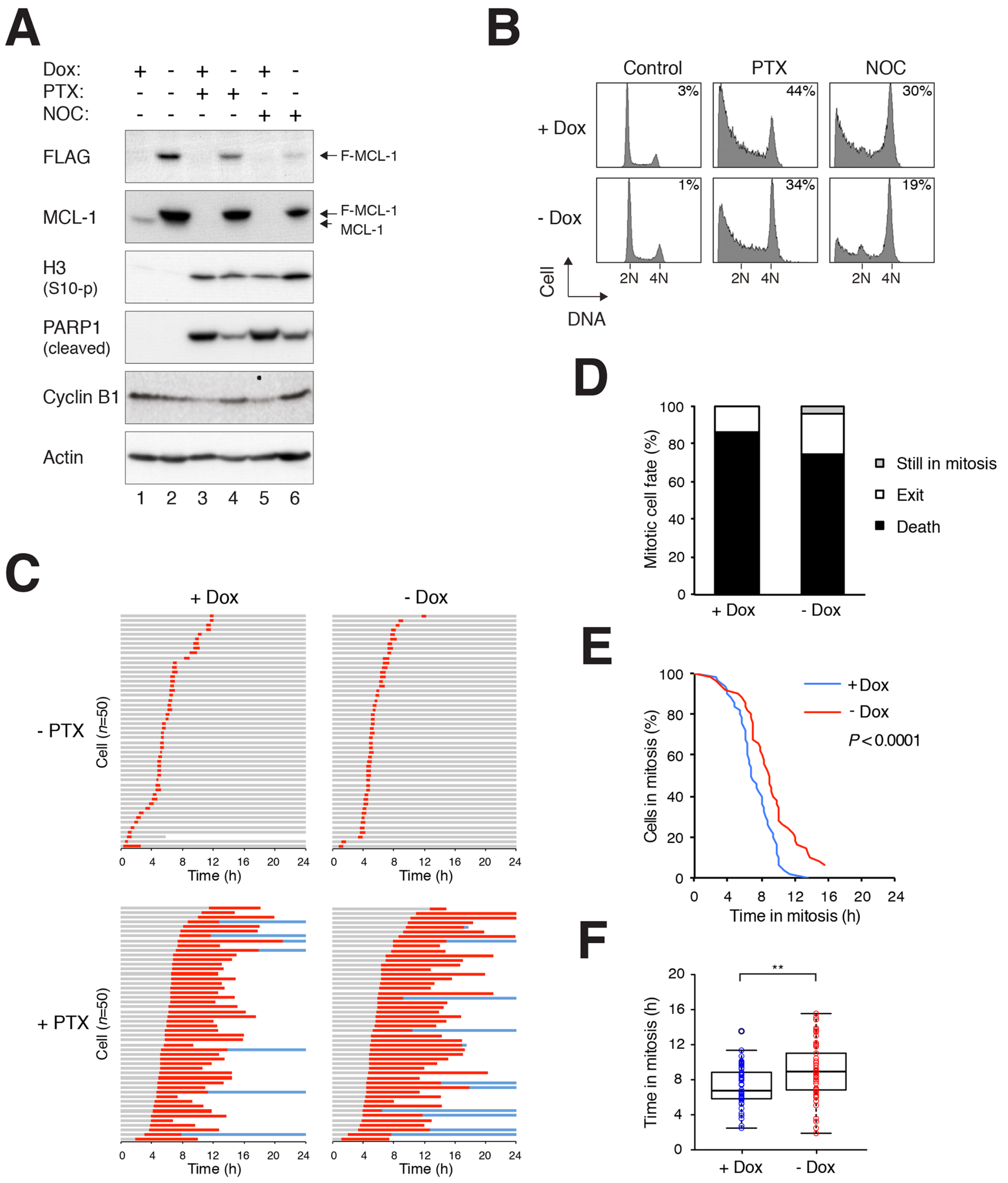

Supplementary Figure S9: Delay of mitotic cell death by ectopic expression of MCL-1. A. MCL-1 inhibits PARP1 cleavage. HeLa cells expressing FLAG-MCL-1 were grown in the presence or absence of Dox for 3 days to turn off or on FLAG-MCL-1 respectively. The cells were exposed to PTX (31.3 ng/ml) or NOC (25 ng/ml). After 24 h, the cells were harvested and analyzed with immunoblotting. Note that for as unknown reasons FLAG-MCL-1 was detected by the anti-FLAG antibodies weakly, the FLAG-MCL1 and endogenous MCL-1 (which is absent during mitosis) were also detected with anti-MCL1 antibodies. **B.** MCL-1 inhibits apoptosis. Cells were treated as in (A). After 24 h, the cells were harvested and analyzed with flow cytometry. The percentages of sub-G_1 cells are quantified. **C.** MCL-1 delays mitotic cell death. HeLa cells expressing MCL-1 (and also histone H2B-GFP) were grown in the presence or absence of Dox for 3 days to turn off or on MCL-1 respectively. The cells were synchronized, exposed to PTX (31.3 ng/ml) and analyzed with live-cell imaging (*n*=50). Key: same as Figure 1C. **D.** Percentage of different mitotic fates from (C). **E.** The durations of mitotic block of PTX-treated cells from (C) are plotted using Kaplan-Meier estimator. **F.** Box-and-whisker plots showing elapsed time from mitotic entry to either mitotic exit or death (cell still in mitosis at the end imaging were included). Student's t-test $^{**}P<0.01$.

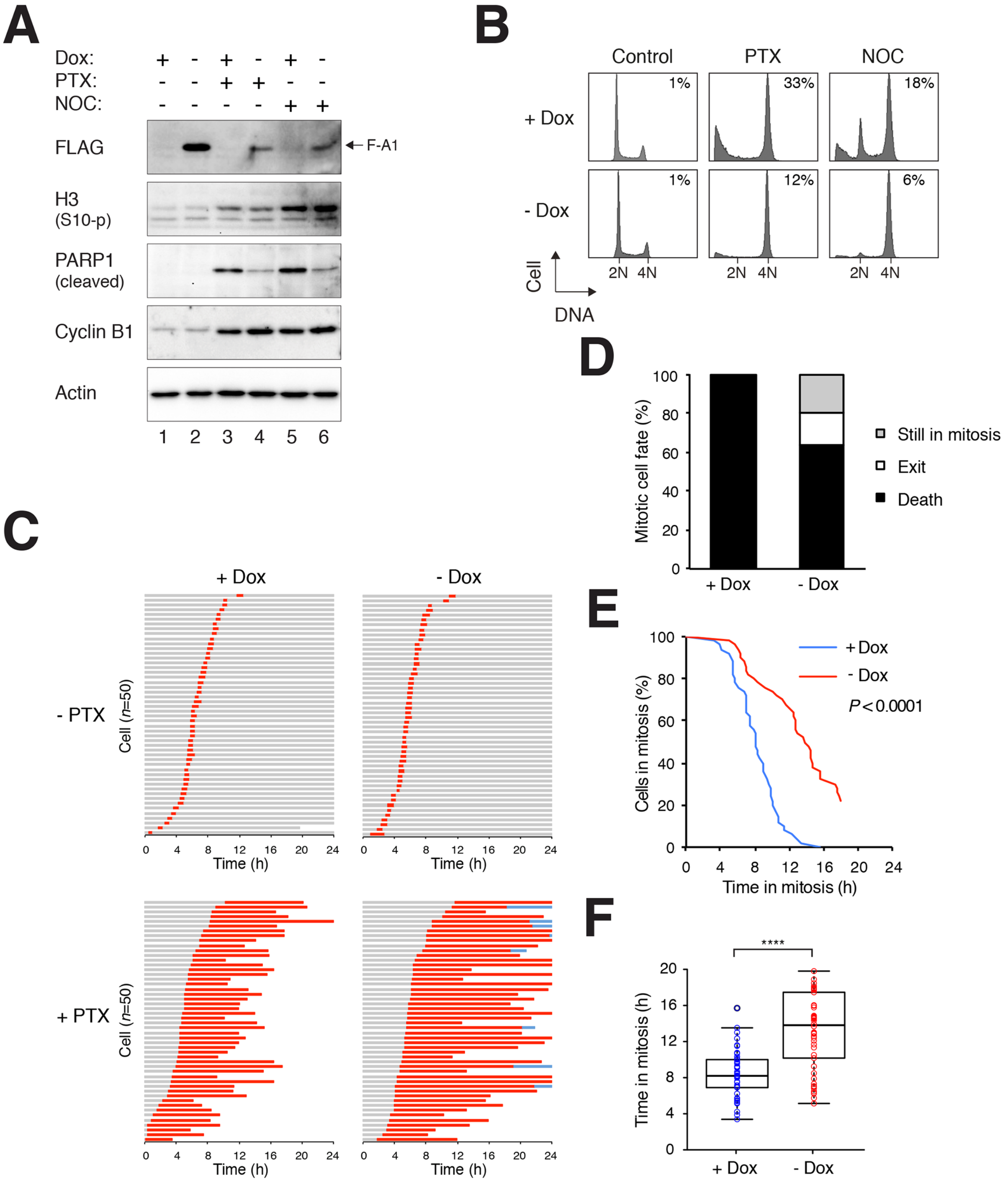

Supplementary Figure S10: Delay of mitotic cell death by ectopic expression of A1. A. A1 inhibits PARP1 cleavage. HeLa cells expressing FLAG-A1 were grown in the presence or absence of Dox for 3 days to turn off or on FLAG-A1 respectively. The cells were exposed to PTX (31.3 ng/ml) or NOC (25 ng/ml). After 24 h, the cells were harvested and analyzed with immunoblotting. **B.** A1 inhibits apoptosis. Cells were treated as in (A). After 24 h, the cells were harvested and analyzed with flow cytometry. The percentages of sub-G_1 cells are quantified. **C.** A1 delays mitotic cell death. HeLa cells expressing A1 (and also histone H2B-GFP) were grown in the presence or absence of Dox for 3 days to turn off or on A1 respectively. The cells were synchronized, exposed to PTX (31.3 ng/ml) and analyzed with live-cell imaging (*n*=50). Key: same as Figure 1C. **D.** Percentage of different mitotic fates from (C). **E.** The durations of mitotic block of PTX-treated cells from (C) are plotted using Kaplan-Meier estimator. **F.** Box-and-whisker plots showing elapsed time from mitotic entry to either mitotic exit or death (cell still in mitosis at the end imaging were included). Student's t-test ****P<0.0001.

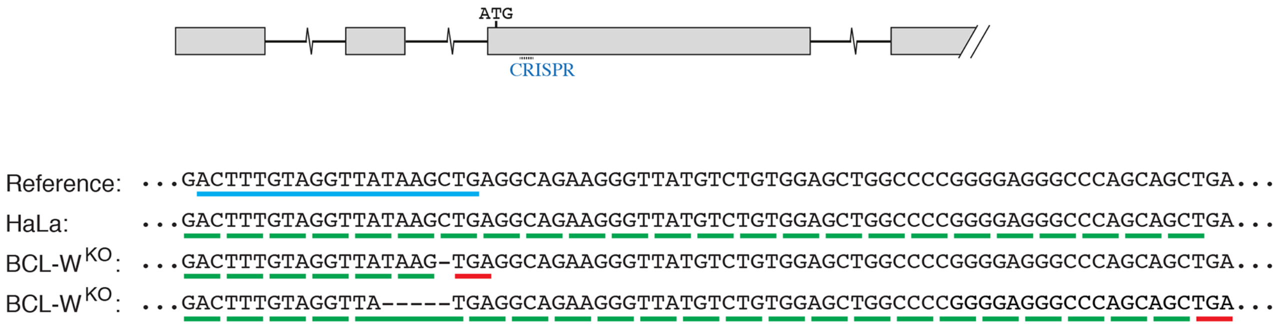

Supplementary Figure S11: Disruption of *BCL-W* gene. Schematic diagram of the *BCL-W* gene is shown at the top (boxes: exons; lines: introns). ATG indicates the position of the start of the ORF; and CRISPR indicates the position of the targeting site of the CRISPR-Cas9 construct. The sequence of normal *BCL-W* according to the reference human sequence database is shown. The targeting sequences of the CRISPR-Cas9 are highlighted in blue. The *BCL-W* gene from HeLa and BCL-W^{KO} cells was sequenced. The reading frame of the ORF is indicated (green). Deletions of one and five bases were found in BCL-W^{KO}, resulting in frame shifts and premature termination (red). These changes are expected to produce products containing 22 aa and 37 aa, respectively (full length BCL-W contains 193 aa).

Supplementary Video S1: Normal mitosis. HeLa cells expressing histone H2B-GFP were synchronized at G_1/S with a double thymidine procedure and released into the cell cycle in the absence of PTX. The cells were analyzed with time-lapse microscopy. An example of normal mitosis is shown (left: bright field; right: histone H2B-GFP).

See Supplementary File 1

Supplementary Video S2: Mitotic cell death. HeLa cells expressing histone H2B-GFP were synchronized at G_1/S with a double thymidine procedure and released into the cell cycle in the presence of PTX. The cells were analyzed with time-lapse microscopy. An example of mitotic cell death is shown (left: bright field; right: histone H2B-GFP).

See Supplementary File 2

Supplementary Video S3: Mitotic slippage. HeLa cells expressing histone H2B-GFP were synchronized at G_1/S with a double thymidine procedure and released into the cell cycle in the presence of PTX. The cells were analyzed with time-lapse microscopy. An example of mitotic slippage is shown (left: bright field; right: histone H2B-GFP).

See Supplementary File 3
